# Intratumoral Immunotherapy and Tumor Ablation: A Local Approach with Broad Potential

**DOI:** 10.3390/cancers14071754

**Published:** 2022-03-30

**Authors:** Zachary J. Senders, Robert C. G. Martin

**Affiliations:** Department of Surgery, Division of Surgical Oncology, University of Louisville, Louisville, KY 40202, USA; zachary.senders@louisville.edu

**Keywords:** intratumoral immunotherapy, immuno-oncology, ablation, irreversible electroporation, oncolytic virus, radiofrequency ablation, microwave ablation, cryoablation

## Abstract

**Simple Summary:**

The goal of immuno-oncology is to potentiate a durable antitumor immune response. The immunosuppressive tumor microenvironment (TME) presents a substantial challenge to current systemic therapies due in part to a lack of available tumor antigen, dense stroma, an abundance of immunosuppressive cells and cytokines, and poor antigen presentation. A multimodal approach that combines intratumoral immunotherapy with tumor ablation addresses several of these challenges. In this review, we evaluate the current data regarding this promising therapeutic approach.

**Abstract:**

Several intratumoral immunotherapeutic agents have shown efficacy in controlling local disease; however, their ability to induce a durable systemic immune response is limited. Likewise, tumor ablation is well-established due to its role in local disease control but generally produces only a modest immunogenic effect. It has recently been recognized, however, that there is potential synergy between these two modalities and their distinct mechanisms of immune modulation. The aim of this review is to evaluate the existing data regarding multimodality therapy with intratumoral immunotherapy and tumor ablation. We discuss the rationale for this therapeutic approach, highlight novel combinations, and address the challenges to their clinical utility. There is substantial evidence that combination therapy with intratumoral immunotherapy and tumor ablation can potentiate durable systemic immune responses and should be further evaluated in the clinical setting.

## 1. Introduction

Immuno-oncology (IO) is a cornerstone of modern cancer therapy. Most notably, checkpoint inhibitors (CI) are now first-line treatment for a variety of metastatic solid tumors, and their role in the adjuvant and neoadjuvant settings is expanding. There are, however, some limitations to existing systemic IO, as efficacy is largely dependent on an antigen-specific effector T-cell-mediated response that may not occur in the setting of a poorly antigenic tumor. Effector cells must also overcome several physical and biochemical obstacles in order to exert their effector function. Dense tumor stroma, an inhibitory chemokine/cytokine profile, and the presence of a variety of immunosuppressive cell types contribute to a harshly immunosuppressive tumor microenvironment (TME). These barriers are not easily surmounted with checkpoint inhibitor therapy alone. There is now widespread interest in developing new therapeutic modalities that can transform a cold, immunosuppressive TME into one that is immunogenic [1].

Human intratumoral immunotherapy (HIT-IT) is one promising strategy that contends with the drawbacks of systemic IO while addressing the TME directly [2,3]. HIT-IT represents a broad category of therapies with variable mechanisms of action, though all are based on direct delivery of the therapeutic agent by local injection into the tumor [4]. There are several advantages of this approach. First, HIT-IT is not affected by the pharmacokinetic limitations of systemic administration. Agents therefore can be injected locally at a concentration many times higher than would be tolerated systemically, while allowing for fewer systemic toxicities. Local injection also opens the door to novel therapies that would be impractical to administer systemically. Second, HIT-IT can induce antigen presentation and immune cell priming. This is particularly important in poorly immunogenic tumors. Such a mechanism is distinct from that of CIs, which generally rely on the activation of pre-existing antigen-specific effector cells. Third, HIT-IT can circumvent the deranged mechanisms of immune cell trafficking and migration that are often induced by an immunosuppressive TME. Further, therapeutics can be designed to induce recruitment of immune cells to the TME if not deliver immune cells to the tumor directly. A major goal of treatment with these local therapies is induction of an immune-mediated effect in non-injected tumors, or the so-called anenestic (e.g., abscopal) effect [2,4]. Unfortunately, the anenestic effects seen in in most human trials of HIT-IT have thus far been modest at best [5,6]. For example, the most well-studied and currently only FDA-approved HIT-IT, Talimogene laherparepvec (T-VEC), can induce rapid regression of injected melanoma lesions but only induces a limited response in distant disease [7]. Additionally, the need for repeated injections has limited its application to easily accessible tumors.

Tumor ablation is another modality that has gained recent interest for its potential to induce an antitumor immune response. Ablative techniques vary in mechanism; however, their therapeutic efficacy is primarily achieved via local tumor destruction. Ablated tumor tissue is subsequently left in situ where inflammatory mediators, damage-associated molecular patterns (DAMPs), and other immunomodulatory factors are released [8]. Importantly, there is a large source of tumor antigen that remains in situ and available for processing by antigen-presenting cells [9]. While ablation has been shown to induce a weak anenestic effect in certain tumor types such as hepatocellular carcinoma [10], the immunostimulatory effect of ablation alone is generally insufficient to produce a durable antitumor immune response. This has prompted interest in enhancing the immune effects of ablation by combining it with IO [11]. Preclinical studies have been conducted to examine the addition of CIs to tumor ablation [12], and while data have been promising [13], this approach still suffers from the limitations inherent to systemic IO.

**Figure 1:**
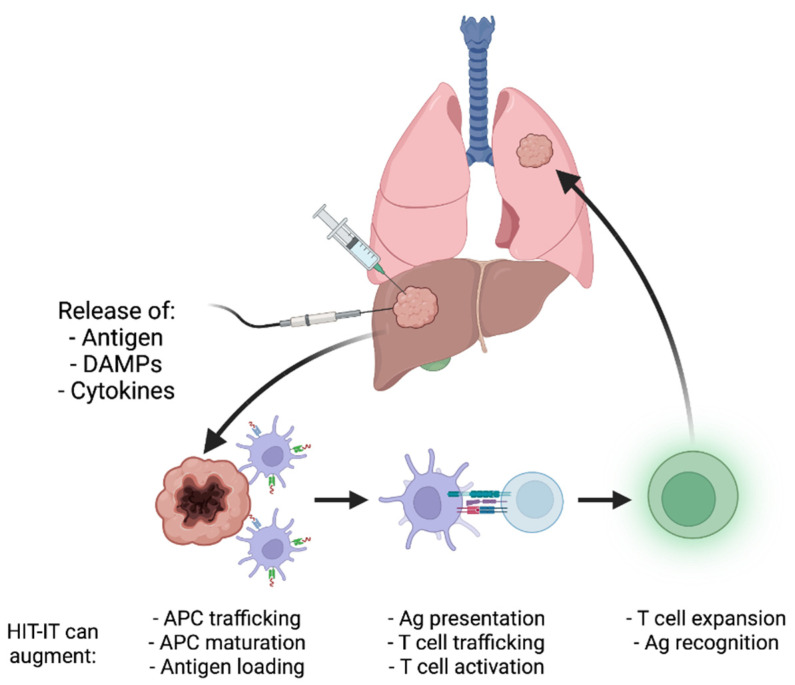
Schematic theoretical overview of the process by which intratumoral immunotherapy and tumor ablation may induce anenestic immune effects and durable antitumor immune responses. Ablation induces tumor destruction, which provides an antigen source for antigen-presenting cells (APCs). APCs then present tumor antigen to effector cells that migrate to other sites of disease and potentiate immune responses. Intratumoral therapeutics may augment one or more of these processes.

As the immunomodulatory effects of tumor ablation have been better defined and novel HIT-IT are developed, it has become clear that these modalities could have unique synergistic potential (Figure 1) [8]. In this article, we will review the existing data regarding combination HIT-IT and tumor ablation (Table 1), summarize ongoing clinical trials, and discuss future directions for this promising multimodal therapeutic approach.

## 2. Therapeutic Strategies for Combination HIT-IT and Tumor Ablation

### 2.1. Virus-Based Therapies

Oncolytic viruses have been in various stages of investigation for over a century. While these agents were initially investigated for their direct oncolytic potential, recent interest has centered on their ability to induce antitumor immunity. T-VEC, a genetically modified herpes simplex type 1 virus, was the first oncolytic virus to gain FDA approval (2015) and is indicated in the treatment of advanced melanoma. T-VEC has been shown to induce regression of injected tumors and can potentiate an immune-mediated response in non-injected lesions; however, it is ineffective for control of distant disease [7]. Various strategies have been proposed to augment the immune effects of T-VEC, most notably by adding CI; however, results from a phase 3 clinical trial were disappointing [14]. Combination therapy with oncolytic viruses and tumor ablation is another approach under investigation. Several preclinical studies have been published that highlight their potential synergies.

Sun et al. [15] investigated Alphavirus M1 in combination with irreversible electroporation (IRE) in a pancreatic adenocarcinoma mouse model, and showed that the virus enhanced therapeutic efficacy of IRE and induced a systemic T cell immune response. Pancreatic adenocarcinoma is notoriously immunosuppressive due in part to a dense tumor stroma. Interestingly, the authors of this study found that virus entry into tumor cells was enhanced by the porosity of the cell membrane induced by IRE. They also suggest that IRE had a stroma-modifying effect, which allowed better access of the virus to tumor cells within the TME. While there are some limitations to the clinical use of this particular oncolytic virus, this study illustrates that IRE, with its unique mechanism of action, can produce changes in the TME to enhance viral infection of tumors that would otherwise be resistant due to dense stroma.

Multimodal treatment with oncolytic viruses and tumor ablation is theoretically counterproductive, as ablative therapies are designed to destroy tumor cells while oncolytic viruses require host tumor cells to be living in order to replicate. Investigators have attempted to address this challenge in novel ways. Yamada et al. [16] used a neoadjuvant approach by first treating tumors with oncolytic herpesvirus G47delta followed by radiofrequency ablation. They used this approach in a hepatocellular carcinoma mouse model and showed that the combination produced a robust CD8+ T-cell-dependent immune response in both primary and contralateral tumors. Mice treated with the combination therapy were also able to resist tumor rechallenge, suggesting lasting tumor specific immunity. The staged treatment approach in this study allowed time for the virus to infect viable tumor cells and replicate before those cells were destroyed via RFA. While the exact mechanism of synergy is not fully described by the authors, it is known that oncolytic viruses enhance antigen presentation and priming of T cells. The subsequent inflammatory reaction caused by RFA may then promote trafficking and infiltration of the effector cells that were previously primed due to viral oncolysis. Additionally, APCs that have infiltrated the periphery of the TME after viral therapy would then be available to process antigen released after RFA. More robust mechanistic studies are needed to better understand the additive effects of these modalities.

### 2.2. Cell-Based Therapies

Adoptive cell immunotherapy (ACT) has shown clinical efficacy in treating hematologic malignancies and is under active investigation for use in solid tumors. Multiple late-phase clinical trials are underway to evaluate tumor-infiltrating lymphocyte therapy (TILS) in metastatic melanoma, and other cell-based immunotherapies are in early trials. Of the many challenges limiting the usefulness of ACT in treating other solid tumor types, several of these may be addressed by combining intratumoral injection of immune cells with tumor ablation. First, cells delivered intravenously must traffic to and infiltrate tumor tissue to exert their effector functions, which is a process that is often inhibited by poorly immunogenic tumors. Immune cells delivered intratumorally can circumvent the physical barrier created by dense tumor stroma, do not need to traffic hematogenously, and could be delivered in sufficient quantities to overcome immunosuppressive signaling in the TME [17]. Tumor ablation could further enhance the intratumoral infiltration of ACT by both breaking down the tumor stroma and by destroying immunosuppressive cells in the TME before the therapeutic cells are delivered. Second, many poorly immunogenic tumors do not produce sufficient antigen to generate a robust native immune response. Adoptive transfer of antigen presenting cells (APC) has thus shown little efficacy in treating these tumors given a lack of sufficient in situ antigen for processing. Tumor ablation, however, could be used to generate large quantities of antigen that would be immediately available for processing by intratumorally injected APCs [18].

Approaches that combine intratumoral ACT with tumor ablation have been studied in the preclinical setting. Machlenkin et al. [19] used cryotherapy with injection of dendritic cells in mouse models of lung cancer and melanoma. They showed that although the dendritic cell treatment alone increased the proliferation of CD8+ T cells, only the combination therapy produced effector memory cells. Mice treated with the combination therapy survived longer and were more resistant to subsequent tumor rechallenge, indicating a durable response. One notable aspect of their study design, and a potential major advantage of this approach, is their use of immature unloaded dendritic cells. Current dendritic cell ACT protocols most often employ mature dendritic cells that are loaded with tumor antigen or are genetically modified ex vivo, adding significant complexity. By contrast, the dendritic cells used in this study were essentially antigen loaded in vivo as a result of the cryotherapy. An approach such as this, which does not require antigen loading, is much more translatable and could save significant time and cost. Further, mature, antigen-loaded DCs may be less able to cross-present antigens and induce antigen spreading, which is critical to a durable immune-mediated response [20].

Other groups have expanded on the intratumoral DC and ablation strategy by investigating agents that can augment antigen processing and effector T cell activation. Udagawa et al. [21] briefly incubated DCs with Bacillus Calmette-Guerin cell wall skeleton (BCG CWS), which is known to act as a Toll-like receptor agonist, before intratumoral injection in a murine metastatic colon cancer model. An increase in tumor infiltrating CD8+ T cells was seen in mice treated with incubated DCs compared to immature DCs, in addition to substantially greater tumor regression and resistance to tumor rechallenge. These findings indicate that perhaps stimulated but antigen-naïve DCs are most optimal for use in HIT-IT/ablation protocols, rather than DCs that are either fully immature or antigen-loaded. Further work in this area was performed by Nakagawa et al. [22], who developed a novel agent, OK432, derived from Group A streptococcus, which stimulates DCs via TLR3, TLR4, and B2 integrin. They investigated the use of OK432-stimulated DCs in combination with RFA for hepatocellular carcinoma (HCC), based on earlier studies using trans-arterial liver-directed therapy. The efficacy of OK432-stimulated DCs in preclinical studies was impressive, and subsequently a first-in-human randomized phase I/II clinical trial was conducted [23]. In this trial, 30 patients were randomized to an intratumoral injection of either immature DCs or OK432-stimulated DCs at the time of RFA. Patients treated with stimulated DCs had significantly longer recurrence-free survival (24.8 vs. 13.0 months, *p* = 0.003), though overall survival was not significantly different. Notably, DCs were harvested from patients’ peripheral blood, incubation and expansion took only one week, and the treatment was well tolerated by patients. Another study showed similar antitumor immune responses with direct IT injection of OK432 in a breast cancer RFA model [24]. It remains to be determined whether in vivo DC stimulation with OK432 is as effective as ex vivo stimulation as part of an ACT protocol. Regardless, these positive findings warrant further study in humans.

While there is a clear theoretical basis for combining ablation with APC-based therapies, the role of other cell-based therapies is not well defined. Few studies have evaluated non-APC immune cells in combination with ablation. In a phase I study, Lin et al. [25] investigated allogenic NK cells in combination with IRE for the treatment of locally advanced pancreatic adenocarcinoma. Progression-free and overall survival were modestly improved with the combination therapy. Alnaggar et al. [26] conducted a similar phase 1 study in stage 4 hepatocellular carcinoma and showed a comparatively modest survival benefit. There is an overall lack of data in this area; however, combination therapy with non-APC intratumoral ACT and ablation does not appear to have substantial efficacy.

### 2.3. Pattern Recognition Receptor (PRR) Agonists

Pattern recognition receptors, primarily expressed by antigen-presenting cells, have long been known for their role in regulating the inflammatory response to pathogens. PRRs act to recognize both conserved pathogen-associated molecular patterns (PAMPs) in the setting of infection as well as endogenous cellular damage-associated molecular patterns (DAMPs) released by stressed or dying cells. Upon recognition of PAMPs or DAMPs, PRRs potentiate a wide variety of immunologic effects including cytokine release, APC differentiation, antigen presentation, and lymphocyte activation. Several vaccine adjuvants have been successfully developed that take advantage of these immunostimulatory properties [27]. There has also been significant interest in developing PRR agonists that can potentiate immune-mediated antitumor responses [28]. Systemic administration of PRR agonists, however, has not proven effective. These agents are poorly tolerated, especially at doses high enough to overcome their poor bioavailability. Systemic administration also does not recapitulate the complex local process that occurs via PRR signaling at the site of pathogenic infection or cell damage. These challenges may be addressed with intratumoral delivery of PRR agonists, which has shown some limited efficacy in the preclinical setting. A multitude of clinical trials evaluating intratumoral PRR agonist monotherapy are ongoing.

There is clear synergistic potential in combining intratumoral PRR agonists with ablation. By delivering PRR agonists to the site of ablation, they may function as an in situ vaccine adjuvant, potentiating the immune response to antigen released by ablation. Behm et al. [29] evaluated this concept by combining RFA and intratumoral injection of CpG-ODN, which is a Toll-like receptor (TLR) 9 agonist and has been used as a pathogenic vaccine adjuvant in humans. Their study showed that HCC bearing rabbits treated with RFA and CpG-ODN survived significantly longer, with less distant metastases, and resisted tumor rechallenge better than animals treated with either agent as monotherapy. Interestingly, treatment with CpG-ODN alone caused an upregulation of both Th1 immunostimulatory cytokines such as IL-2 and IFNy, and immunosuppressive Th2 cytokines such as IL-10, which tended to produce a tolerogenic immune phenotype overall. RFA, however, induced the release of DAMPs such as heat shock proteins which, combined with CpG-ODN, promoted a robust primarily Th1 immune response. Another study by van Brok et al. [30] produced similar results using cryoablation and TLR9 agonist in a murine melanoma model, and provided insight into the mechanism of synergy. Ablation alone caused DCs in the tumor draining lymph nodes to become antigen-loaded, but the addition of TLR9 agonist caused an increase in CD80 expression, indicating DC maturation, and an increase in number of DCs, indicating proliferation. Additionally, only DCs from mice in the combination group were able to efficiently cross-present antigens to MHC-restricted T cell subsets. These and other data from Shankara et al. [31] suggest that ablation causes release of antigen and DC loading, which is necessary to produce downstream immunologic effects, but is not sufficient without additional immunostimulatory signaling. This may explain the weak abscopal effects seen clinically after ablation, but provides a rationale for further development of adjunctive immune stimulators.

Although TLR agonists are promising agents to combine with tumor ablation, they can cause notable off target effects. Various TLRs are known to be expressed on certain tumor types and T cell subsets. Signaling through receptors on these cell types can promote tumor proliferation and immune evasion. Additionally, variable downstream effects are seen dependent on the type of TLR targeted. For example, TLR4 agonism enhances proliferation and activity of immunosuppressive regulatory T cells, while TLR1/2 and TLR8 agonism reduces the suppressive effects of Tregs [32]. Balancing the complex downstream effects of TLR agonists is clearly a major barrier to their clinical potential.

Other PRR pathways are being evaluated for their therapeutic potential. STimulator of INterferon Genes (STING) is an intracellular PRR that recognizes cytosolic DNA and stimulates the production of type 1 interferons. The STING pathway plays a central role in endogenous antitumor innate immunity primarily through host immune cell ingestion and recognition of tumor-derived DNA. Activation of this pathway induces production of type 1 interferons which regulate widespread local and systemic immune functions including DC maturation, T cell priming, chemotaxis, and further cytokine production [33]. STING agonists have shown an antitumor effect in preclinical studies [34]; however, significant systemic toxicities have been seen with therapeutic doses when delivered intravenously. Early-phase clinical trials have thus focused on combining lower doses of systemic STING agonist with checkpoint inhibitors [35] or delivering the agent intratumorally. As the immunostimulatory effects of these agents occur initially in the cytosol of DCs in the TME, a novel approach to enhancing their effect has been to combine them with IRE. One theoretical basis for this combination is that IRE permeabilizes the cell membrane and allows the anionic STING agonists to enter the tumor cells. DCs then phagocytose dying STING agonist-loaded tumor cells at which point the pathway is stimulated. Several studies have evaluated combination STING/IRE and have confirmed that they are potently synergistic. Go et al. [36] used a murine lung cancer model to demonstrate that combination STING/IRE resulted in an increase in activated DCs, CD4+ and CD8+ T cells, and M1 macrophages in the TME, and a decrease in M2 immunosuppressive macrophages. Data from Lasarte-Cia et al. [37] showed similar results in melanoma and HCC models. Both studies reported substantial tumor regression in the combination group. Based on these interesting preliminary data and a clear mechanistic basis for their synergy, combination STING/IRE deserves more investigation.

### 2.4. Biomaterials

Synthetic biomaterials are increasingly being studied for use as immune modulators and many of these novel materials are uniquely suited to local delivery. Several of these agents have been studied as adjuncts to tumor ablation. Zhou et al. [38] developed a mannose-derived carbon dot nanoparticle that facilitates tumor antigen delivery to APCs. They showed that these nanoparticles efficiently captured antigen and DAMPs released after microwave ablation in a mouse model of HCC. After capturing antigen and damage signals, the nanoparticles targeted DCs, stimulated DC maturation, and led to enhanced T cell activation. The combined treatment causes significant tumor regression and resistance to tumor rechallenge. Another novel approach by Yu et al. [39] involved the use of magnetic nanoclusters loaded with an indoleamine 2,3-dioxygenase (IDO) inhibitor. IRE, when delivered after injection of the nanoclusters, induced local magnetic fields, which enhanced direct tumor destruction and triggered release of the IDO inhibitor. The result was an increase in CD8+ T cells and a decrease in Tregs infiltrating the tumor. These studies are important proofs of concept that advanced biomaterials may have a role in cancer therapy, and can be designed to augment existing ablative therapies.

Smaller molecule synthetic biomaterials have also been evaluated in combination with ablation. Chen et al. [40] combined a rho-associated kinase (ROCK) inhibitor, which stimulates DC phagocytosis, with a hydrogel polymer that traps tumor antigen after RFA. The polymer caused an increase in DC antigen uptake and a gradual release of ROCK inhibitor in the cytoplasm, which resulted in tumor regression and an increase in survival in a murine melanoma model. Yang et al. [41] engineered a therapeutic based on a double-emulsified lipoxidase and hemin that, when injected after RFA, continuously produced cytotoxic lipid radicals while using tumor debris as fuel. These so-called tumor-killing, tumor-fueled nanoreactors caused more efficient residual local tumor destruction and also induced antitumor immunity across multiple murine cell lines. Another promising biomaterial, IP-001, has been described by Korbelik et al. [42] and is unique in that it was engineered specifically to be combined with ablative therapies. This variant of N-dihydrogalactochitosan, when injected peritumorally and combined with subsequent ablation, has been shown to induce an antitumor immune response via multiple mechanisms. The polymer causes antigen sequestration and recruitment and stimulation of APCs, which induces a Th1 T cell immune response. IP-001 has a promising side effect profile and is under active investigation in early-stage human trials.

### 2.5. Cytokines

Intratumoral cytokine injection has also been studied in combination with tumor ablation. Granulocyte macrophage colony-stimulating factor (GM-CSF) is a cytokine that, among other functions, activates APCs and has been shown to trigger a systemic antitumor response. This cytokine is a critical part of the mechanism of action of T-VEC, which incorporates a GM-CSF cassette, causing local expression in injected tumors [7]. Interestingly, in early studies of T-VEC, local tumor destruction was similar between virus constructs with and without GM-CSF cassettes; however, the viruses that included GM-CSF induced significantly more regression in non-injected tumors. Conversely, tumors treated with GM-CSF alone did not produce a robust response. The underlying mechanism of T-VEC therefore is reliant on both direct viral oncolysis as well as GM-CSF-mediated APC stimulation. Studies that combine GM-CSF or other cytokines with tumor ablation produce antitumor immune effects through an analogous mechanism. Chen et al. [43] combined intratumoral GM-CSF microspheres with microwave ablation and CTLA-4 blockade and demonstrated a tumor-specific antitumor immune response mediated by CD4+, CD8+, and NK cells. Lemdani et al. [44] combined RFA with a GM-CSF-BCG hydrogel, which induced primary tumor regression in a murine colorectal carcinoma model, and completely eradicated microscopic secondary lesions. Larger distant secondary lesions regressed when PD-1 inhibitor was added. Others have investigated IL-2 [45], IL-7, and IL-15 [46] in combination with ablation and have shown some success in animal models. In a novel approach, Johnson et al. [47] combined RFA with an intratumoral injection of tumor-specific monoclonal antibodies conjugated to IL-2. This treatment induced tumor regression and immunologic memory in a murine colon cancer model.

## 3. Opportunities and Challenges

Multimodality therapy with HIT-IT and tumor ablation presents unique opportunities and challenges. Substantial progress has been made in investigating the efficacy of this therapeutic approach in the preclinical setting, yet few translational studies have been completed thus far and results of those that have been completed have been underwhelming. Questions remain regarding the role that this approach will play in increasingly complex and effective IO treatment paradigms that are emerging. Additionally, many promising current IO therapies have been woefully understudied for their use as HIT-IT or in combination with ablation. As new IO agents are developed, it will be important to consider their potential for use as HIT-IT or in combination with ablation, especially if systemic delivery is found to be toxic or infeasible.

There is obvious potential for therapies to be developed de novo to exploit the individual mechanism of action of each ablation technique and the unique biologic properties of the resultant ablated tumor. For example, thermal ablation tends to induce necrotic cell death, which produces significantly more inflammation, immunostimulatory cytokine release, and available tumor antigen compared to the apoptosis induced by IRE [8,48]. Some data, however, suggest that apoptosis induces better T cell priming and thus a more potent antitumor immune response [49]. IRE has also been shown to induce a larger area of infiltrative immune cells [50], higher systemic levels of IL-6 [51], and attenuation of systemic Tregs [52]. A better understanding of the immunogenic cell death pathways and other immunologic effects that are promoted by each ablation modality would allow investigators to combine them with HIT-IT that are more complementary and avoid modalities that produce an immunosuppressive cell death pathway [53]. For example, oncolytic viruses that are known to promote autophagy and apoptotic cell death could be pared with a thermal ablation technique that promotes necrosis, thereby inducing multiple modes of immunogenic cell death. Strategies could also be developed to leverage the cell membrane permeability caused by IRE, in a similar fashion to combination STING/IRE. Other intracellular PRRs would be well suited for such an approach. Poly-ICLC, for example, is an agonist of intracellular TLR3 and has been shown to have efficacy in preliminary studies in combination with IRE [54]. While the majority of current IO agents are directed towards cell surface proteins, IRE could be utilized to target novel intracellular pathways, thereby substantially broadening the scope and utility of new IO therapies.

While avoidance of systemic toxicity is a key advantage of local therapy with HIT-IT and ablation, the addition of systemic adjuncts such as checkpoint inhibitors could further enhance the resultant systemic immune response induced by this therapeutic strategy. Checkpoint blockade has proven to be of limited efficacy in poorly immunogenic tumors, especially tumors of an immune desert phenotype [1]. These tumors are characterized by low tumor mutational burden, low numbers of antigens, and poor T-cell priming, leading to an overall lack of tumor-infiltrating effector T cells (TIL). An approach that utilizes combination HIT-IT and ablation to promote TIL infiltration in an otherwise poorly immunogenic tumor might also benefit from the addition of checkpoint inhibition [48]. Positive results from early phase studies of tumor ablation combined with checkpoint inhibition lend support to this strategy [55]. Few clinical trials exist investigating both HIT-IT and ablation in combination with systemic therapy. A study from the Netherlands, however, using IRE, nivolumab, and an intratumoral TLR9 agonist was opened in 2020 and is currently recruiting (PANFIRE-3) [56]. More complex multimodality therapies, although challenging to implement, are theoretically possible. For example, the large number of TIL that are generated after combination cryotherapy and intratumoral dendritic cell injection could subsequently be harvested and used as a source of cells for adoptive transfer. Though such a strategy is technically challenging today, immuno-oncology is trending toward a heavily multimodal approach that will only expand in scope and complexity as additional therapies become clinically available.

There are several challenges to implementing combined HIT-IT and ablation therapeutic approaches [57]. Melanoma is well-suited for HIT-IT not only because it is highly immunogenic, but also because the common sites of origin and metastasis are amenable to repeated local therapies without invasiveness. In contrast, access to some tumors (e.g., visceral tumors) can require significantly more invasive methods that make repeated injections or sequential treatment protocols impractical. The HIT-IT agents used in such a setting therefore must be potent enough to be effective as a single dose treatment and must be effective when delivered in the same setting as ablation [58]. Fortunately, there has been substantial progress in interventional radiology techniques such that most solid tumors can be safely accessed percutaneously [59]. Additionally, with the wider availability of IRE, some tumor types that were traditionally not amenable to thermal ablation techniques can now feasibly be ablated. Further development of such techniques would expand the scope and utility of combination HIT-IT and ablation therapy.

Another practical challenge to studying combination HIT-IT and ablation is that most patients undergoing visceral tumor ablation are doing so for curative intent. Patients that have no evidence of viable disease after treatment would lack the in situ untreated metastatic lesions that are often used to evaluate anenestic response to HIT-IT. It would be exceedingly challenging to determining radiographic response in a primary tumor that has been both injected with HIT-IT and ablated, especially given the changes that are typically seen radiographically after tumors are ablated. itRECIST criteria [60] were developed specifically for use in trials of HIT-IT to address the limitations in applying existing RECIST criteria to these patients [61]. No existing framework including itRECIST, however, is suitable for studying treatment response in a solitary primary tumor treated with combination HIT-IT and ablation. Novel, valid, potentially non-radiographic methods of evaluating treatment response in this setting (e.g., ctDNA) are needed. Clinical trials in patients with metastatic disease could be an alternative; however, preclinical data will need to be compelling in order to justify subjecting these patients to an invasive ablation procedure that is outside the standard of care. It is also not clear if data from studies in patients with metastatic disease can be extrapolated to those with local disease, given the important biological dissimilarities of primary versus metastatic tumors. Despite the challenges, patients with metastatic disease are especially likely to benefit from an anenestic immune response induced by combination HIT-IT and ablation.

## 4. Conclusions

A multimodal approach with HIT-IT and tumor ablation addresses many of the major limitations of current IO-based therapies, and has the potential to induce a durable antitumor immune response. Further studies, especially of a translational nature, are needed to determine which patients, which combination of modalities, and in which tumor types this approach is most beneficial.

## Figures and Tables

**Table 1:** Selected studies investigating intratumoral immunotherapy in combination with tumor ablation.

Category of IT Immunotherapy	Agent	Ablation Technique	Species Studied	Tumor Type	Author
Cell-based therapies	DCs	RFA	Mouse	Urothelial	Dromi
	DCs	Cryo	Mouse	Lung/Melanoma	Machlenkin
	DCs	Phototherapy	Mouse	Colon/Melanoma	Saji
	DCs + BCG	Cryo	Mouse	Colon	Udagawa
	OK432-stimulated DCs	RFA	Human	Hepatocellular	Kitahara
	OK432-stimulated DCs	RFA	Mouse	Colon	Nakagawa
	NK cells	IRE	Human	Hepatocellular	Alnaggar
	NK cells	IRE	Human	Pancreas	Lin
	NK cells	IRE	Human	Pancreas	Lin
Pattern recognition receptor agonists	TLR3 agonist (Poly-ICLC)	IRE	Mouse/Rabbit	Hepatocellular	Vivas
	TLR7 agonist (1V270)	IRE	Mouse	Pancreas	Narayanan
	TLR9 agonist (CpG-ODN)	Cryo	Mouse	Melanoma	den Brok
	TLR9 agonist (IMO-2125)	IRE	Human	Pancreas	Geboers
	TLR9 agonist (CpG B)	RFA	Rabbit	Hepatocellular	Behm
	STING agonist (c-di-GMP)	IRE	Mouse	Melanoma/Hepatocellular	Lasarte-Cia
	STING agonist (RR-CDA)	IRE	Mouse	Lung	Go
Oncolytic viruses	Human Adenovirus Type 5 (rhAd5)	RFA	Human	Hepatocellular	Xie
	Human HSV Type 1 (G47d)	RFA	Mouse	Neuroblastoma	Yamada
	Human HSV Type 1 (G47d)	RFA	Mouse	Hepatocellular	Yamada
	Alphavirus M1	IRE	Mouse	Pancreas	Sun
Biomaterials	IDOi-loaded nanoclusters	IRE	Mouse	Prostate	Yu
	Carbon dots	MWA	Mouse	Hepatocellular	Zhou
	N-dihydrogalactochitosan (IP-001)	MWA	Mouse/Human	Various	Korbelik
	HLCaP nanoreactors	RFA	Mouse	Breast/Colon/HCC/Melanoma	Yang
	Thermogel + ROCK inhibitor	RFA	Mouse	Melanoma	Chen
Cytokines	GM-CSF-BCG hydrogel	RFA	Mouse	Colon	Lemdani
	GM-CSF microspheres	MWA	Mouse	Hepatocellular	Chen
	IL-2 microspheres	MWA	Mouse	Hepatocellular	Wu
	KS-IL2	RFA	Mouse	Colon	Johnson
	IL-7/IL-15	RFA	Mouse	Breast	Habibi
Others	DC stimulant (OK432)	MWA	Mouse	Breast	Li
